# International nursing advanced competency-based training for intensive care: a europe-wide survey

**DOI:** 10.1186/2197-425X-3-S1-A920

**Published:** 2015-10-01

**Authors:** R Endacott, C Jones, S Blot, C Boulanger, M Ben-Nun, K Iliopoulou, I Egerod, MJ Bloomer

**Affiliations:** Monash University, School of Nursing and Midwifery, Frankston, Australia; Plymouth University, School of Nursing and Midwifery, Plymouth, United Kingdom; University of Liverpool, Liverpool, United Kingdom; Ghent University, Department of Internal Medicine, Ghent, Belgium; Royal Devon and Exeter Hospital, Exeter, United Kingdom; Kaplan Medical Centre, Rehovot, Israel; Athens Military Hospital, Athens, Greece; Copenhagen University Hospital, Copenhagen, Denmark

## Introduction

The role of the ICU nurse is becoming increasingly complex and Intensive Care nursing (ICN) is regarded as a highly specialised area of nursing. Across Europe pre-registration nursing programs have some consistency, however there is no consensus on education or practice requirements for ICN despite increasing clinical demand.

## Objectives

The objectives of this study were to map adult ICN training programs throughout Europe; examine what competency based training has been developed for ICN, and review current educational structures and process to enable possible barriers to a Europe-wide competency-based training program to be identified.

## Methods

Modelled on the CoBaTrICE study [1], a survey was distributed through ICN networks throughout Europe to collect data on current ICN training and education, methods of assessment/ accreditation and regulatory frameworks/guidelines that inform ICN education and training.

## Results

Survey data was collected in 2014. Thirty-two responses were received, representing 24 countries in Europe. Whilst most countries (83%) reported presence of national ICN society, ICN was recognised as a specialty area in only 54% of countries. ICN education was provided in 66% of countries, across a mix of settings (50%) and at university (25%), resulting in a variety of qualifications. Prior experience in ICU was required in 43.8% of countries prior to formal ICN education, and programs ranges in duration from 240 hours to 2 years. Regardless of whether formal ICN education was provided, most countries reported challenges: lack of national standard, lack of time and lack of protection for the title.

## Conclusions

Regardless of the WHO ICN curriculum [2], there remains considerable variation across Europe in terms of education, certification, regulation and scope of practice for specialist ICN nursing roles. This highlights the need for standardisation to reduce role confusion, enable mobility of the ICN workforce and promote equivalence in advanced ICN practice roles.

## Grant Acknowledgment

ESICM
